# Adapting Scenario Planning to Create an Expectation for Surprises: Going Beyond Probability and Plausibility in Risk Assessment

**DOI:** 10.1111/risa.70112

**Published:** 2025-09-18

**Authors:** James Derbyshire, Mandeep Dhami, Ian Belton, Dilek Önkal, Terje Aven

**Affiliations:** ^1^ University of Chester Chester UK; ^2^ Middlesex University London UK; ^3^ Strathclyde University Glasgow UK; ^4^ Northumbria University Newcastle UK; ^5^ University of Stavanger Stavanger Norway

**Keywords:** plausibility, probability, risk assessment, scenario planning, surprise, uncertainty

## Abstract

The need for risk assessments to take full account of uncertainty by going beyond probability and creating an expectation for surprises has recently been highlighted in this journal. This paper sets out an adaptation to the Intuitive Logics (IL) scenario‐planning method that assists risk assessors to achieve this aim. We demonstrate the effectiveness of this adaptation through a controlled experiment. The controlled experiment took the form of a simulated IL scenario‐planning exercise in which individuals assigned values representative of extreme outcomes to sets of simple and more complex clusters of driving forces under three experimental conditions representing alternative uncertainty expressions (“probable,” “plausible,” and “surprising”). The values assigned in the “probable” and “plausible” conditions were not significantly different from each other. However, the “surprising” condition resulted in the assignment of more extreme values than either of the other two conditions. The complexity of a set of clustered driving forces had no effect. A follow‐up analysis showed that participants interpreted the words “probable” and “plausible” similarly. This is problematic for scenario methods like IL, which are claimed to stretch consideration of the future's potential extremity beyond what it would be using probability by instead employing plausibility. If participants interpret “probable” and “plausible” similarly, then using plausibility instead of probability will not stretch their thinking as desired. By adapting IL in the simple way this paper outlines, scenario planning can assist risk assessors to go beyond both probability and plausibility, thereby taking fuller account of uncertainty and improving anticipation of surprises.

## 1 Introduction

The need for risk assessments to take full account of uncertainty by going “beyond probability” has been extensively studied in the literature (see, e.g., Flage et al. 2014; Paté‐Cornell 2012; Aven 2014). Along similar lines, Derbyshire and Aven (2025) recently argued that developing an expectation for surprises as part of a risk assessment is key to their avoidance. This requires that official risk assessments make the potential for surprises explicit, not only by going beyond probability but also by taking account of “unknowledge” through “structured acts of imagination” (Derbyshire and Aven 2025). Structured acts of imagination can be enabled by scenario planning, which is the focus of this paper.

Humans routinely see the future as a continuation of the past. Yet, while the future can indeed sometimes simply be a continuation of present trends, discontinuous jumps are also common and play a critical role in shaping the future (Lempert et al. 2002; van Notten et al. 2005). Even if they are analogous to past events, at least in terms of the extremity of their impact, surprises can far surpass what was conceivable based on the knowledge available up to the point of their occurrence. These characteristics make it difficult for risk assessors to conceive of their possibility. This is especially so if assessors exclusively employ standard risk‐analysis methods that focus attention on what is known and can be empirically evidenced, thereby directing attention away from more extreme and novel possibilities that are much harder to evidence (Derbyshire and Aven 2025; Lempert et al. 2002).

This paper addresses that problem. It adapts the popular scenario‐planning method known as Intuitive Logics (IL) to create an expectation for surprises in the form of extreme outcomes. It tests this adapted scenario‐planning method using a controlled experiment, the results of which demonstrate how a consideration of surprising extremes can be stimulated. This adapted approach to scenario planning can be used as a complement to more traditional risk‐analysis methods to ensure risk assessments take full account of uncertainty by going beyond probability (and even plausibility) and considering surprises. The outlined scenario approach is one means by which to operationalize the consideration of surprises within an official risk assessment, the need for which was established by Derbyshire and Aven (2025).

In addressing this issue of surprises, the paper discusses several related matters. Through its experimental findings, the paper provides evidence supporting the suggestion of Glette‐Iversen et al. (2022) that plausibility should be seen as a “measure of uncertainty capturing a combination of likelihood and judgments on the supporting knowledge” (Glette‐Iversen et al. 2022, abstract). The outlined experiment shows that, when asked to assign extreme values to the resolved outcomes of a set of related driving forces within an IL scenario‐planning process, participants asked to assign “extreme, yet still probable” and “extreme, yet still plausible” values assign similar values.

This suggests that the concepts of probability and plausibility are perceived similarly. This is unsurprising because the concept of plausibility is based on past outturns and known possibilities, just as both frequency‐based (i.e., “objective”) probability and the Bayesian variety of subjective probability are, as evident in their central requirement to list all possibilities in advance. This requirement embeds thinking in knowns established through past occurrences. This similarity between plausibility and probability is problematic for IL scenario planning. The use of plausibility in IL scenario planning is claimed to make it more suitable for considering novel, emergent, and extreme possibilities in the form of surprises and discontinuities (Derbyshire 2017, 2022). Yet, questions about its ability to do this have been raised for some time (e.g., van Notten et al. 2005). For instance, it could be suggested that plausibility is perceived by individuals in a similar way to probability, meaning IL scenario planning would not stretch thinking beyond past occurrences as intended.

The plan for this paper is as follows: Section 2 briefly highlights the discussion already underway in this journal for some time regarding the need to take fuller account of uncertainty in risk assessments by going beyond probability and considering surprises. Section 3 describes the standard IL scenario‐planning method. Section 4 examines the nature of the concept of plausibility on which IL is based. Section 5 describes our experimental study and Section 6 describes its method. Section 7 presents its results and Section 8 discusses them. Section 9 makes some concluding remarks, drawing out the practical implications for the use of IL scenario planning as part of a comprehensive risk assessment.

## 2 The Need to Go Beyond Probability in Risk Assessments

Risk assessments traditionally employed probabilities to express uncertainties. However, contemporary risk‐assessment frameworks recognize the need to “go beyond probability” (e.g., Flage et al. 2014). There are several reasons that overly focusing on probability in a risk assessment might increase susceptibility to surprises.

An assigned probability is based on a set of knowledge available at the time of its assignment. This set might be incomplete or deficient in some other way and therefore lack “strength” (Derbyshire and Aven 2025).^1^ A low probability might therefore be assigned to a highly impactful extreme outcome based on a set of presently available knowledge that lacks strength. If so, it would be unwise to focus on the probability alone and to dismiss the possibility of the highly impactful extreme outcome on that basis. To do so would increase susceptibility to being surprised by the extreme outcome.

The strength of knowledge is inevitably low for many situations that could have extreme instances because the extremes would result from presently unknown second‐order effects and emergent outcomes that are idiosyncratic and novel. For example, there is much potential for presently unknown second‐order effects in relation to climate change, meaning that the climate's sensitivity to carbon emissions could be far greater than currently thought (Derbyshire and Morgan 2022). This in turn means that derived values for the upper limit of the climate‐sensitivity range could greatly underestimate climate change's extremity and full potential impact (Derbyshire and Morgan 2022).

A subjective probability is a belief about a particular outcome (e.g., a particular scale of impact from climate change) based on a set of knowledge, some or all of which might be theoretical rather than empirical. However, the problem with using subjective probability to represent uncertainties is that surprises stem not from knowledge, but its inverse, unknowledge―that is, what we do not presently know and may not be aware of not knowing (Derbyshire and Aven 2025). Subjective probability places the emphasis on what *is* known, which may exacerbate any natural tendency (e.g., confirmation bias) to overlook what is unknown, thereby increasing susceptibility to surprise.

As noted, contemporary risk‐assessment frameworks acknowledge this problem and insist that a probability is never used to express uncertainties in isolation from an assessment of the strength of knowledge on which the probability is founded. An integral part of this assessment of the strength of knowledge is the consideration of unknowledge—that is, knowledge that is not presently available but which would be of importance to assessing the focal risk accurately if it were available (Derbyshire and Aven 2025). Unknowledge is more abstract, difficult to conceive, and easier to overlook than knowledge. For that reason, its consideration―essential to developing an expectation for surprises as part of a risk assessment—requires “structured acts of imagination” (Derbyshire and Aven 2025).

This is where IL scenario planning might contribute. It is designed to stimulate consideration of the factors that could lead to surprisingly extreme outcomes by focusing on plausibility. Yet, we argue it can only do this if adapted in the manner we later set out. For reasons outlined in the next sections, in its common plausibility‐based format, IL scenario planning cannot stimulate consideration of surprises in the form of extreme outcomes.

## 3 The IL Scenario‐Planning Method

The IL scenario‐planning method typically comprises eight stages (see Cairns and Wright 2018). In Stage 1, a focal issue is established. In the experiment outlined in this paper, there were two focal issues. The experiment was conducted during the coronavirus pandemic and so one group of participants considered the potential impact of further waves of coronavirus in the United Kingdom. For another group the focal issue was the development of the housing market in the United Kingdom, which has been subject to extreme outcomes in the past, such as that associated with the Great Crash of 2008 (i.e., the “credit crunch”), which led to the “Great Recession.” We report both sets of results but the illustrative figures in this section are those shown to the group of participants who focused on the impact of coronavirus as a focal issue.^2^

**FIGURE 1:**
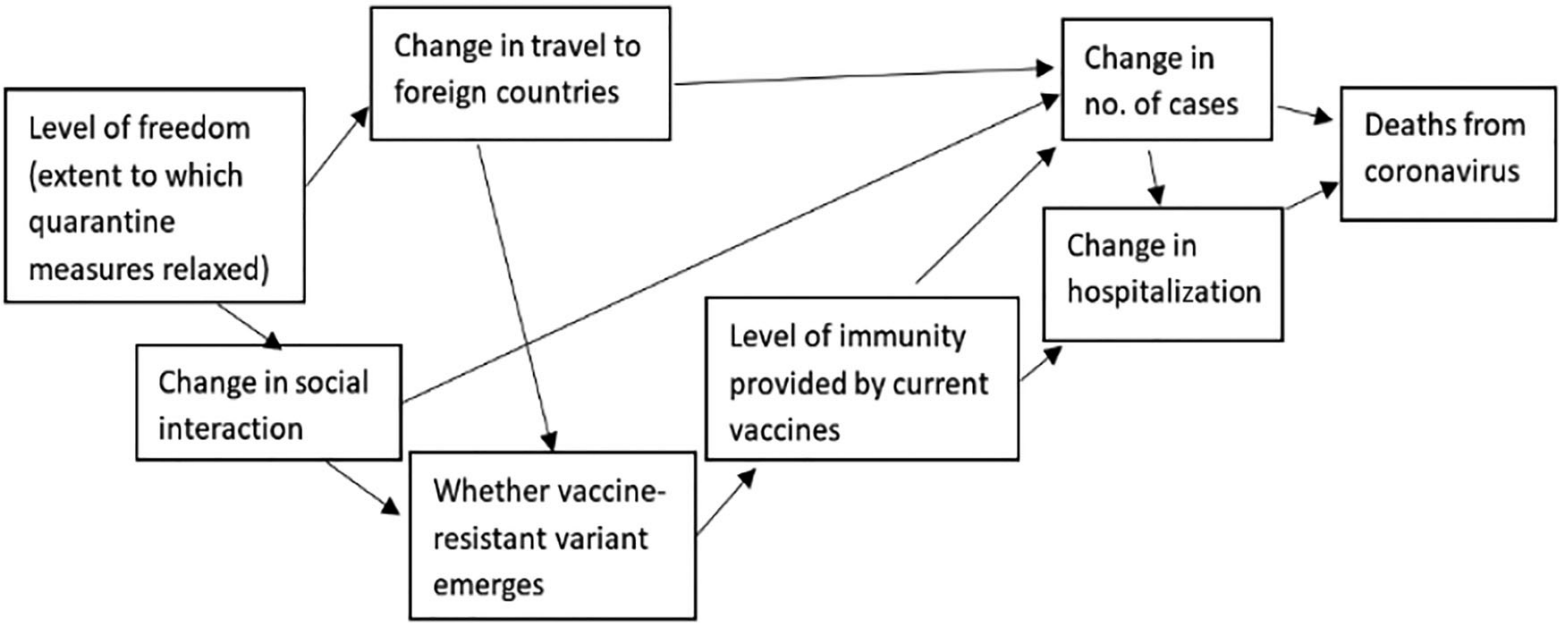
An example of a clustered set of driving forces constructed at Stage 3 of the Intuitive Logics scenario‐planning method.

In Stage 2, a range of individual “driving forces” is identified as being related to the focal issue. In Stage 3, the identified driving forces are related to each other and “clustered” into “influence diagrams” representing the perceived cause‐and‐effect relationships between them, with each cluster leading to a specific “resolved outcome”—for example, deaths from coronavirus in Figure 1. This resolved outcome is one that in turn is believed to have a causal effect on the focal issue. At this stage, clusters are “neutral” in that they display causal links between driving forces but do not make assumptions about the direction of change involved. Thus, in Figure 1, there is no indication as to whether the number of fatalities from coronavirus will increase or decrease.

**FIGURE 2:**
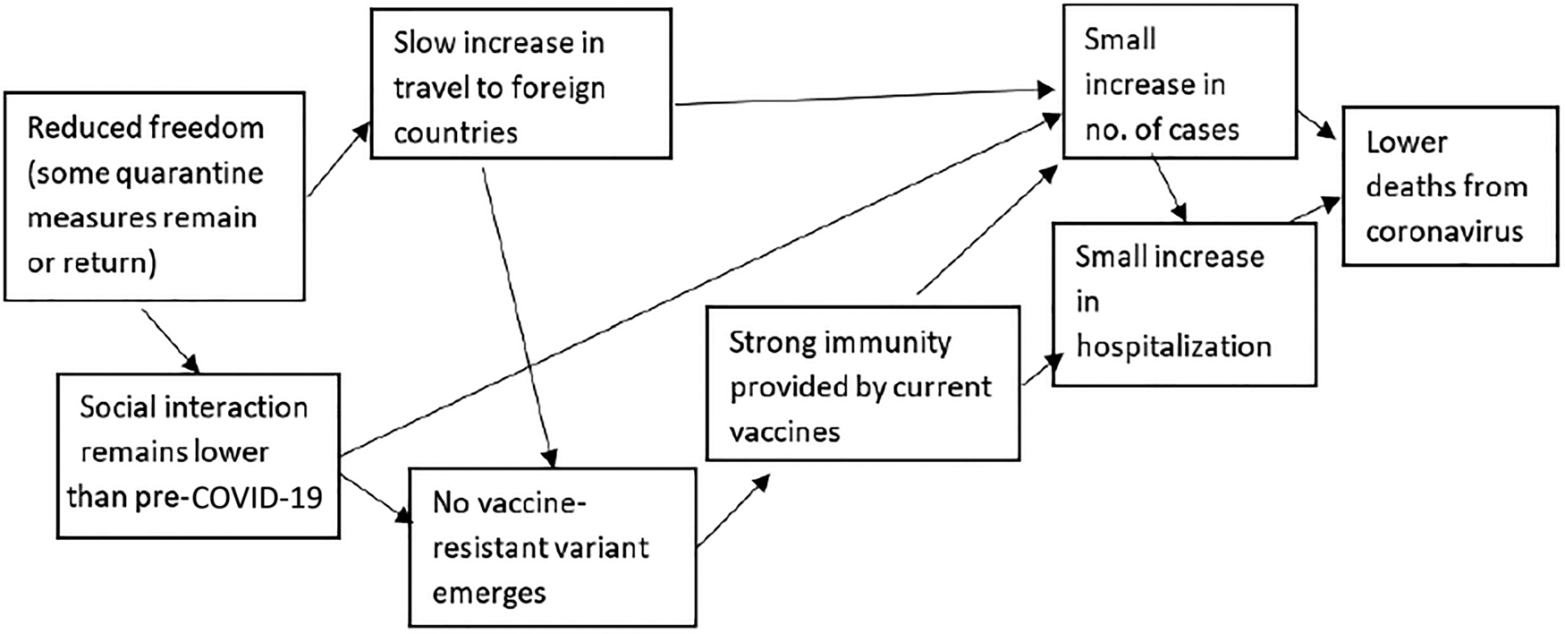
Example of a directional cluster leading to a low resolved outcome.

**FIGURE 3:**
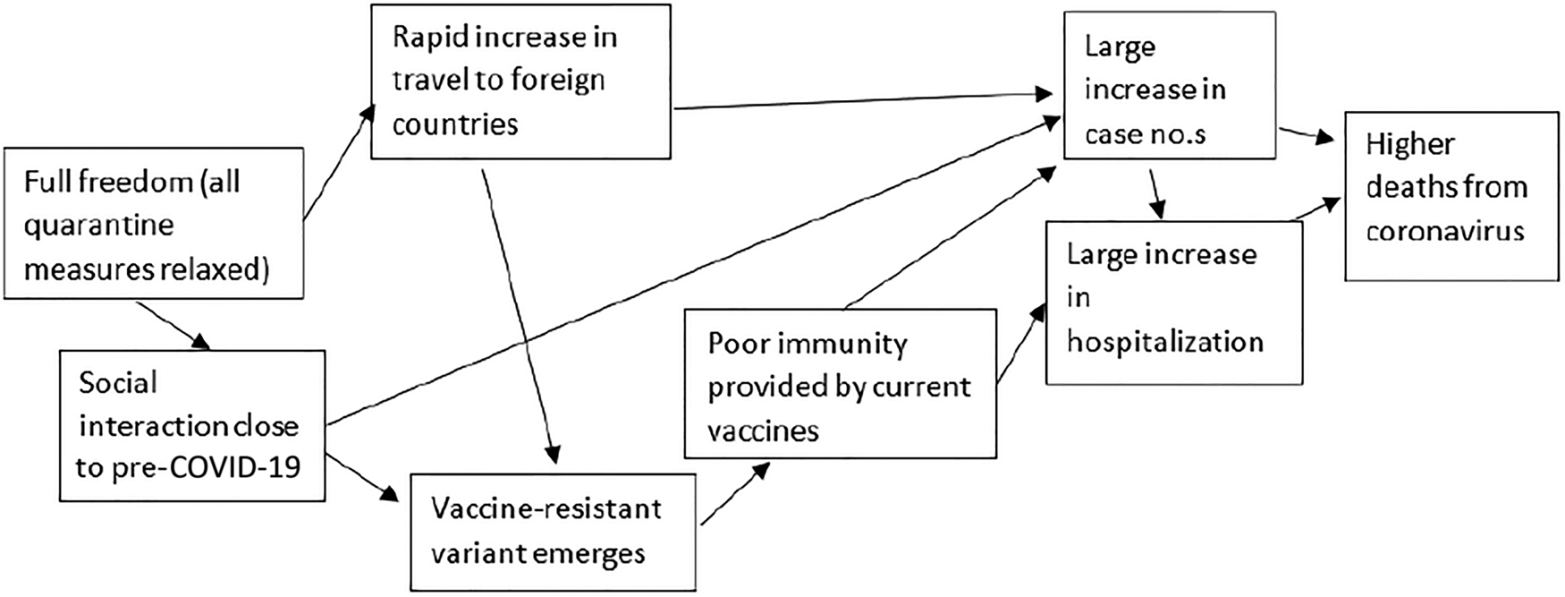
Example of a directional cluster leading to a high resolved outcome.

In Stage 4, two “directional” versions of each cluster are then prepared. These describe alternative ways in which the cluster could resolve itself in the future, leading to “the two most extreme, yet still plausible” resolved outcomes that could occur over the chosen scenario time horizon (Cairns and Wright 2018, 40–41). Note here the emphasis on plausibility.^3^ Outcomes can be expressed as qualitatively contrasting outcomes (e.g., high or low) or as specific values of a given variable, such as the number of fatalities, as in Figures 2 and 3.

In Stage 5, clusters are ranked^4^ in terms of the uncertainty of these resolved outcomes and their impact on the focal issue. The two clusters with the highest perceived uncertainty and impact are selected for further consideration. These two clusters are then labeled A and B, and in Stage 6, they provide the dimensions for a 2 × 2 matrix wherein each quadrant represents one combination of the two extreme yet still plausible resolved outcomes of the selected clusters. In Stage 7, descriptors are added to each quadrant of the 2 × 2 matrix. Finally, in Stage 8, these descriptors are elaborated into a narrative scenario pertaining to each of the quadrants, which describes how its resolved outcomes come about.

This matrix‐based approach is currently the standard method for scenario planning in many fields (Bishop et al. 2007; Derbyshire, Feduzi, et al. 2023; Phadnis et al. 2014; Ramirez and Wilkinson 2014). By asking users to think in terms of plausibility in Stage 4, this method eschews probability to enable consideration of extreme futures (Janasik 2021; Ramirez and Selin 2014; Selin and Guimarães Pereira 2013; Wilkinson et al. 2013; van der Helm 2006). Probability is thought to be too constraining due to its basis in past occurrence and presently available knowledge, both of which may greatly under‐represent the potential extremity of the future (Derbyshire 2022). Yet, as we explore in the next section, there may be much more similarity between plausibility and probability than advocates of scenario planning might like to acknowledge.

## 4 Probability, Plausibility, and Surprise

Urueña (2019) contrasts plausibility with probability, suggesting that plausibility offers a greater scope and flexibility, and a greater adaptability to diverse decision contexts, than does probability. The logic is that “‘The plausible’ subsumes ‘the probable’” (Urueña 2019, 20) and so plausibility enables consideration of a broader range of futures than would probability. Yet, Connell and Keane (2006, 96) maintain that a “scenario, or discourse is plausible if it is conceptually consistent with what is known to have occurred in the past.” Moreover, in their work on the availability heuristic for judging probability, Tversky and Kahneman (1973, 229) state that “The plausibility of the scenarios that come to mind, or the difficulty of producing them…serve as a clue to the likelihood of the event,” which again suggests a link between plausibility and probability.

Furthermore, research shows that plausibility judgments tend to be based “on the relative potential truthfulness of incoming information compared to our existing mental representations” of phenomena (Lombardi et al. 2013, 50; see also Schmidt‐Scheele 2020; Urueña 2019). These existing mental representations stem from past experiences. This is corroborated by Walton et al. (2019, 49), who find that scenario‐planning participants judge the plausibility of a scenario based on “what has gone on before,” explicitly looking to their past experiences as a frame of reference. Relatedly, Chinn and Brewer (2001) show that individuals judging plausibility contrast given statements with their existing knowledge about a phenomenon, ranging from fact‐based background knowledge to underlying theories or intuitive gut‐feelings about “how‐it‐works.” Indeed, in their review of the literature on plausibility, Glette‐Iversen et al. (2022, abstract) concluded that plausibility is a “measure of uncertainty capturing a combination of likelihood and judgments on the supporting knowledge,” again suggesting some crossover with probability in its subjective form. Others have reached similar conclusions (e.g., Janasik 2021; Morgan and Keith 2008). Therefore, plausibility would appear to embed thinking in past outturns and presently available knowledge in the same way that probability might.

One response to this problem is to employ a catch‐all category of “other” in risk assessments (Kaplan and Garrick 1981). This would reflect and capture the fact that the considered possibilities are based on presently available knowledge, and therefore, cannot be exhaustive—that is, that there will inevitably be some possibilities, presently unknown, which will only emerge and become known over time. This “other” category would include “all the scenarios we have thought of, and also an allowance for those we have not thought of” (Kaplan and Garrick 1981, 17). Yet, simply acknowledging that there are outcomes, or extremes of outcome, about which we are currently unaware does not by itself prevent us from being surprised by them. The point of a risk assessment must surely be to develop awareness of as many possibilities as possible. Only that way can we avoid being surprised by them.

One purpose of a risk assessment must therefore be to shift as many of the possibilities that might otherwise make their way into this category of “other” out of that category and into that of the known possibilities that are explicitly considered by the risk assessors. In other words, to expand the space of possibilities, and the “knowledge landscape” (Derbyshire and Aven 2025) from which it is derived, as widely as possible to capture and render explicit as many of its possibilities as we can.

For the reasons outlined in this section, we suggest that the use of plausibility in IL scenario planning will not help with this essential task of expanding the range of considered possibilities because it embeds thinking in presently available knowledge (van der Heijden 2005), much as would the use of probability. Plausibility bounds perception of future possibilities within existing knowledge and pre‐existing assumptions, inhibiting proper consideration of extremes and surprises. This might explain the observed tendency to focus on the most easily evidenced aspects of the future in scenario planning (MacKay and McKiernan 2004). If this is correct, then using plausibility in scenario planning may not assist risk assessors in creating an expectation for surprises as part of a risk assessment.

The concept of surprise is the near inverse of plausibility; it focuses thinking on contrasts with past experiences and present knowledge (Teigen and Keren 2003) rather than embedding expectations within these. When experienced via a novel empirical observation, surprise results from the contrast between the difference or extremity of the observation and an individual's pre‐existing representation of the range of potential extremity based on past occurrence (Maguire et al. 2011). Surprises are essentially violated expectations (Kahneman and Miller 1986; Simandan 2020). Whereas plausibility is based on fit with presently available knowledge, surprise is based on a contrast with it. A similar contrast can be drawn between surprise and probability because probability, like plausibility, is grounded in pre‐existing knowledge and experience (Glette‐Iversen et al. 2022; Urueña 2019).

If, as suggested above, surprise results from a contrast between the extremity of an observation and an individual's pre‐existing representation of the range of potential extremity, then the avoidance of surprises must involve stretching the representation of the range of potential extremity so that it captures the full potential extremity of the future. We therefore suggest that explicitly using the uncertainty expression “surprising” instead of “plausible” or “probable” as a prompt for assigning outcome values to clustered sets of driving forces in Stage 4 of IL will extend participants’ consideration of potential future extremes beyond past occurrences or what is conceivable based on presently available knowledge.

## 5 The Present Study

Our experimental study, therefore, focuses on Stage 4 of the IL scenario‐planning process. We ask participants to attribute two extreme and “surprising,” or “plausible,” or “probable” values to the resolved outcome from clustered sets of driving forces. We include “probable” in our test to gauge the extent to which the use of the expression “plausible” as a prompt stretches thinking beyond probability, as assumed by the IL community of users.

Our choice of dependent measure is informed by past research that tested the effect of scenario planning on participants’ perception of the level of uncertainty surrounding the future value of a focal variable. Specifically, in earlier studies on scenario planning's effectiveness (Schoemaker 1993; Kuhn and Sniezek 1996), participants were instructed to provide a point estimate representing a business‐as‐usual value at a certain future date for a specific variable.^5^ In addition, they were asked to provide a 90% confidence interval around that estimate (i.e., a high and a low value between which they believed the true value had a 90% probability of occurring). These measures were all taken from individuals before and after they each completed a simulated scenario‐planning exercise. In both Schoemaker (1993) and Kuhn and Sniezek (1996), the width of participants’ confidence intervals was interpreted as evidence for their perceived uncertainty about the business‐as‐usual point estimate.

**FIGURE 4:**
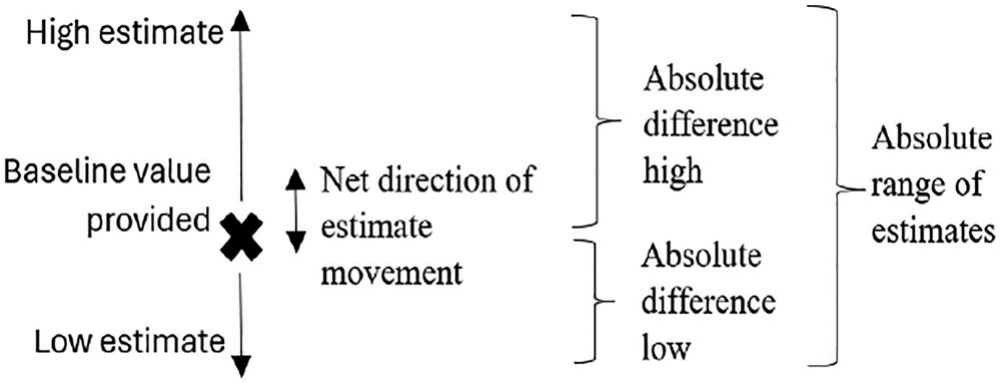
Illustration of dependent measures*. *The middle and right‐hand‐side measures are computed from participants’ low and high estimates. “Net direction of estimate movement” is computed as “Absolute difference (high)” minus “Absolute difference (low).” “Absolute range of estimates” is the sum of “Absolute difference (high)” and “Absolute difference (low).”

In the present study, we also elicit high and low values, but with three variations: (1) participants were given a current (i.e., baseline) value for each focal variable rather than making their own future business‐as‐usual point estimate; (2) in addition, rather than being asked for a 90% confidence interval, participants were asked to generate high and low estimates based on the three different uncertainty expressions/prompts (i.e., surprising, plausible, probable); and (3) we express our main dependent measure in terms of the *absolute range in estimates*. Finally, we conduct exploratory analyses on measures of the absolute distance or movement *up* from the baseline value and *down* from the baseline, as well as the net direction of estimate movement (see Figure 4).

Based on the preceding discussion, we hypothesize that there will be a statistically significant difference in the effect of the three different uncertainty expressions (i.e., surprising, plausible, probable) on the *absolute range in* participants’ estimates (H1). Specifically, we predict that the range will be significantly greater in the “surprising” condition than in either the “plausible” (H1a) or the “probable” (H1b) conditions. We also expect that the range will be greater in the “plausible” than in the “probable” condition (H1c).

In addition, we test the effects of the three uncertainty expressions in the context of complex and simple sets of clustered driving forces. The complexity of the causal relationships leading to a resolved outcome (see Figures 1, 2, 3) is predicted to affect the range of estimates. Specifically, we hypothesize that more complex clusters will generate a *wider absolute range in estimates* compared to simpler clusters (H2). This is because there may be more ambiguity and volatility in the effects of multiple interconnected forces (which will be depicted in a more complex cluster of driving forces), resulting in greater unpredictability or uncertainty. The more intermediate causes there are, as reflected in a more complex cluster of driving forces, the greater the uncertainty surrounding the cluster's resolved outcome, and therefore, the wider the range of the cluster's potential values.

The effect of the interaction between uncertainty expression and causal complexity is more difficult to predict. There are reasons to expect less difference between the effects of the three expressions under a more complex cluster of driving forces than a simple one. This is because a greater number of causal conjunctions and any causal loops, as reflected in a more complex cluster, increases the possibility for compounded second‐order effects and positive feedback, which may take the resolved outcome far beyond its historic values. If a participant successfully traces through the complex causal logic, she may therefore assign more extreme values to its resolved outcome under all uncertainty expressions. Conversely, however, we might just as easily expect a greater difference between the effects of the three expressions from a simpler cluster of driving forces, with the greatest difference being between “surprising” and the other two prompts (i.e., “plausible” and “probable”). This is because greater complexity may make it more difficult for an individual to trace through the causal logic, leading them to fall back on what they already know based on past occurrences. This might then mean that the “surprising” expression will move participants further away from the baseline value compared to the other two expressions, which would result in little movement. Given this ambiguity, we do not make any a priori predictions about the moderating role of cluster complexity on the effect of the three uncertainty expressions.

Finally, we also explored individuals’ understanding of the three uncertainty expressions by asking them to express the extent to which they agreed with statements defining an outcome as surprising, plausible, or probable. The aim here was to determine if individuals differentiated among these three expressions in predictable ways―that is, according to the definitions of these concepts in the extant literature (e.g., Cairns and Wright 2018; Canter et al. 2003; Connell and Keane 2006; Derbyshire 2017; Ramirez and Selin 2014; Schmidt‐Scheele 2020; Teigen and Keren 2003; Urueña 2019; Wiek et al. 2013).

## 6 Method

### 6.1 Participants

Participation was limited to native English speakers aged 18 or over with an undergraduate or higher university degree or equivalent qualification. According to an a priori power calculation using G*Power 3.1 (Faul et al. 2007), a sample size of 249 would be required to achieve the power of 0.80 needed to detect a medium‐sized main effect of uncertainty expression on participants’ movement from the baseline value (*F* = 0.2), with an alpha of 0.05 (two‐tailed).^6^ We oversampled to allow for possible exclusions due to potential participants failing attention‐check questions or providing incoherent estimates. A total sample of 264 was achieved. Individuals were recruited via the Prolific website and received £3 for participation. The final sample of 264 comprised 135 females, 128 males, and 1 nonbinary/third gender. The mean age of the sample was 36.97 (SD = 12.23, range = 18–70). In terms of participants’ level of education, 26.5% of participants had a master's degree, and 6.1% had a doctorate. Participants recruited via Prolific are not domain experts, as participants in scenario planning would usually be. Moreover, there was a nonrandom, self‐selection element to their recruitment since the participants must explicitly choose to participate via the platform. Both factors could affect the external validity of the findings, reducing their generalizability. However, concerning the second of these factors, Mullinix et al. (2015) conducted a widely cited study that analyzed the treatment effects obtained from 20 survey experiments that were implemented on both a representative population‐based sample and a convenience sample (i.e., a self‐selection sample) of participants taken from Mechanical Turk, which is a platform that is similar to Prolific. Mullinix et al. (2015) found there to be considerable similarity between the treatment effects from the representative population‐based samples and those from the online platform's self‐selected convenience samples. Moreover, both Douglas et al. (2023) and Peer et al. (2022) have found that Prolific provides high‐quality data that is better than that provided by Mechanical Turk and other similar platforms. These various studies suggest that online platforms can and do produce generalizable results.

Furthermore, as described in Subsection 6.4, the participants in our experiments were given a brief individual training session based on a worked example. While this will not have increased their domain expertise, which we expect nevertheless would be more than lay given the applicability and relevance of the two domains (i.e., understanding of pandemic spread and economics of house prices), it did introduce them to scenario planning and the relevant concepts (e.g., “clusters” of driving forces). The participants were, therefore, cognizant of the nature of the exercise and its requirements.

### 6.2 Design

We used a 3 × 2 between‐subjects design, with uncertainty expression (surprising, plausible, probable) and the causal complexity of the clustered set of driving forces (complex, simple) as the two independent variables.

### 6.3 Stimuli

As mentioned, our experiment covered two domains: the coronavirus pandemic and house prices in the United Kingdom. Both are domains in which surprisingly extreme outcomes might occur due to “multiplicative” (Taleb 2022) effects resulting from positive feedback. In the case of a pandemic, this multiplicity is captured in the so‐called “R” number, which represents the number of people the virus is passed on to by an infected person. In the case of house prices, increases (or decreases) are self‐reinforcing because they affect the perceived future value of the property, making purchase more (or less) desirable (Bourassa et al. 2019), which can lead to self‐reinforcing house price increases (i.e., a house price bubble).

We developed clustered sets of driving forces for both domains such that a variable associated with its resolved outcome could be assessed numerically—that is, number of COVID‐19 deaths or median house price for a point in the future. Thus, each participant was presented with three clusters for each of the two domains: a neutral cluster of driving forces, a cluster that resulted in a high resolved outcome value, and one that resulted in a low resolved outcome value (see Appendix 1 and Figures 1, 2, 3 for the stimuli).

Below each neutral cluster was a baseline value for the resolved outcome's variable. For COVID‐19 deaths, the figure was the monthly COVID‐19 deaths recorded in England in August 2021, and for house prices, it was the median UK house price for August 2021. After observing the neutral cluster of driving forces, participants received the two directional versions, the first suggesting a high value for the cluster's resolved outcome, and the second suggesting a low value for this resolved outcome.

Finally, there were two versions of each cluster set: a complex and a simple version containing either greater or fewer driving forces and more or fewer causal links between them, respectively (see Appendix 1). Each participant received one complex cluster set and one simple cluster set, counterbalanced in terms of order and in terms of which domain was complex and which was simple.

### 6.4 Measures and Procedure

The study received ethics approval from the Middlesex University Department of Psychology Research Ethics Committee. Participants first undertook a brief individual training session. This used a worked example in a domain unrelated to the two studied here, which was used to introduce the participants to scenario planning and the relevant concepts (e.g., “clusters” of driving forces). All participants first received a neutral cluster. After examining the neutral cluster and baseline resolved‐outcome value, participants were asked a series of follow‐up questions to bring about active engagement with the stimuli and to assess their comprehension of the cluster's causal content, as well as their perception of its complexity/simplicity.

After the training, participants were shown the high and low, complex and simple clusters, in a counterbalanced way across participants, and asked to respond to the following question: “Based on the causal logic of the cluster and the baseline value of [2989 (COVID‐19 domain)/£264,244 (house price domain)], please give a [surprising/plausible/probable] [high/low] value for the [number of COVID‐19 deaths that will be recorded in England in February/median UK house price for August] 2022”^7^ (see Appendix 1).

Lastly, participants answered some basic demographic questions and an exit questionnaire measuring their understanding of the terms “surprising,” “plausible,” and “probable” (see Appendix 2). The 11 items in the exit questionnaire were chosen from the respective literatures on surprise (items 4, 7, and 11), plausibility (items 1, 3, 8, and 10) and probability (items 2, 5, 6, and 9) (Abendroth and Richter 2021; Schmidt‐Scheele 2020; Teigen and Keren 2003; Urueña 2019). The term used at the start of the questionnaire varied across the three between‐subjects conditions to match the term used in the instructions for the main task in that condition (e.g., participants in the surprise experimental condition responded to the 11 items in the exit questionnaire in terms of their relation to the concept of surprise).

The stimuli and measures were presented online via the Prolific website using a survey constructed in Qualtrics, which included an information sheet, consent form, and debrief at the end. Participants were prevented from completing the experiment on a smartphone. There was no time limit on participation, which took approximately 20 min. A minimum plausible duration for completing the experiment was determined based on Prolific's recommendation of three standard deviations below the mean duration taken by participants: all participants exceeded the minimum plausible duration.

In addition, two attention checks were used. At the beginning of the experiment, participants were told “This survey may take up to 20 min, including some online training, and will require your undivided attention during that time. If you are still interested in completing the survey, please select ‘B’ from the options below.” Participants choosing any option but “B” (i.e., “A,” “C,” or “D”) were excluded. A second attention check question was included as part of the exit questionnaire, which instructed participants to “Please answer 10 for this question.” One participant failed this check, and so their data were excluded from analysis. Finally, a further 19 participants who completed the study were excluded from analysis because they provided obviously incorrect or incoherent sets of high and low estimates—for example, where the lowest estimate was higher than the highest estimate. All exclusions were over and above the final sample of 264.

## 7 Results

### 7.1 Comprehension and Manipulation Checks

Participants correctly answered an average of four out of five comprehension questions on the coronavirus clusters (IQR = 1) and three out of five comprehension questions on the house price clusters (IQR = 2). As required, for the coronavirus domain, participants recognized the clusters’ varying complexity, rating the complexity of the simple cluster as significantly lower (M = 3.61, SD = 1.43; out of a maximum 7) than the more complex cluster (M = 5.11, SD = 1.43), t(262) = 8.46, *p* < 0.001, *d* = 1.04. Similarly, for the house price domain, participants rated the complexity of the simple cluster as significantly lower (M = 4.25, SD = 1.38) than the more complex cluster (M = 5.35, SD = 1.21), *t*(262) = 6.88, *p* < 0.001, *d* = 0.85.

### 7.2 Descriptive Statistics

Tables 1 and 2 present the means (and standard deviations) of the absolute difference between baseline value and the estimated high and low for (1) number of coronavirus deaths and (2) median house prices, in relation to the three uncertainty expressions and two levels of cluster complexity. The final column in each table presents the main dependent measure of interest―that is, the sum of the absolute difference between the baseline value and the low estimate and the absolute difference between the baseline value and the high estimate (i.e., the absolute range of estimates).

**TABLE 1:** Means and standard deviations of absolute difference between baseline value and estimated (high and low) number of coronavirus deaths by uncertainty expression and cluster complexity.

		Absolute difference between baseline value and (high/low) estimate					
		Absolute difference (high)		Absolute difference (low)		Absolute range in estimates	
Uncertainty expression	Cluster complexity	M	SD	M	SD	M	SD
Surprise	Simple	41,095	124,013	1138	772	39,935	124,269
	Complex	12,610	22,546	2490	8913	10,120	20,067
Plausibility	Simple	5693	3814	1661	1010	4032	4116
	Complex	12,012	20,978	1274	772	10,739	21,118
Probability	Simple	6654	8910	1531	933	5123	9052
	Complex	6625	4877	1453	800	5172	4936

**TABLE 2:** Means and standard deviations of absolute difference between baseline value and estimated (high and low) median house prices (£) by uncertainty expression and cluster complexity.

		Absolute difference between baseline value and (high/low) estimate					
		Absolute difference (high)		Absolute difference (low)		Absolute range in estimates	
Uncertainty expression	Cluster complexity	M	SD	M	SD	M	SD
Surprise	Simple	365,776	93,251	191,525	48,272	174,251	133,200
	Complex	403,449	168,354	190,830	53,328	212,619	207,032
Plausibility	Simple	315,797	65,366	215,673	43,852	100,123	93,898
	Complex	310,740	73,472	227,883	46,830	82,857	70,177
Probability	Simple	298,930	32,027	232,517	25,783	66,413	51,816
	Complex	315,986	67,513	230,608	26,814	85,378	82,523

### 7.3 Effect of Uncertainty Expression and Cluster Complexity on Absolute Range of Estimates

Separate univariate ANCOVAs were run for each domain to test the hypothesized effects of uncertainty expression and cluster complexity on the absolute range in estimates, controlling for comprehension score. The covariate (comprehension score) was non‐significant (COVID‐19: *p* = 0.284, house price: *p* = 0.633). In support of H1, in both domains, there was a significant main effect of uncertainty expression on the absolute range in estimates (COVID‐19: *F*[2, 263] = 3.98, *p* = 0.020, η_p_^2^ = 0.03, house price: *F*[2, 263] = 21.64, *p* < 0.001, η_p_^2^ = 0.14).

Specifically, in support of H1a and H1b, post hoc tests revealed that in both domains the absolute range in *surprising* estimates was greater compared to both the absolute range in *plausible* estimates (COVID‐19: *t*[92] = 1.91, *p* = 0.030, *d* = 0.29, house price: *t*[131] = 4.39, *p* < 0.001, *d* = 0.66) and *probable* estimates (COVID‐19: *t*[88] = 2.17, *p* = 0.016, *d* = 0.33, house price: *t*[117] = 5.67, *p* < 0.001, *d* = 0.85). In addition, while post hoc tests revealed support for H1c in the house price domain, this was not the case for the COVID‐19 domain. Specifically, as expected, the absolute range in estimated *plausible* median house prices was greater than the absolute range in estimated *probable* median house prices (*t*(166) = 1.77, *p* = 0.039, *d* = 0.27). In the COVID‐19 domain, however, there was no significant difference in the absolute range in *plausible* and *probable* estimates of COVID‐19 deaths (*t*(123) = 1.28, *p* = 0.102, *d* = 0.19).

Contrary to expectation (H2) there was no significant main effect of cluster complexity on absolute range in estimates in either domain (COVID‐19: *F*[1, 263] = 1.18, *p* = 0.278, η_p_^2^ = 0.01, house price: *F*[1, 263] = 1.27, *p* = 0.261, η_p_^2^ = 0.01). Finally, the uncertainty expression by cluster complexity interaction was also nonsignificant (COVID‐19: *F*[2, 263] = 2.63, *p* = 0.074, η_p_^2^ = 0.02, house price: *F*[2, 263] = 0.95, *p* = 0.387, η_p_^2^ = 0.01).

### 7.4 Exploratory Analyses: Effect of Uncertainty Expression and Cluster Complexity on Extremity of Estimates

**FIGURE 5:**
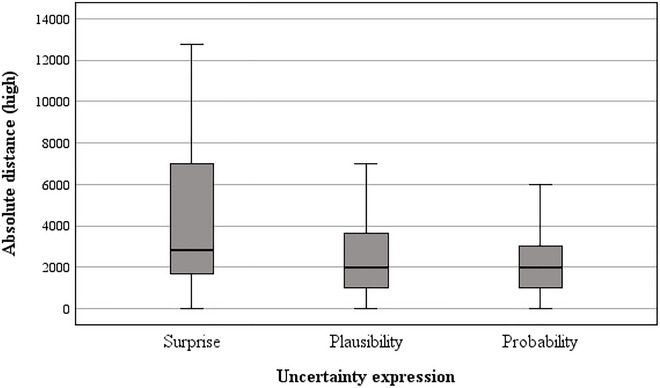
Absolute difference between baseline and *high* estimates for COVID‐19 deaths by uncertainty expression.

**FIGURE 6:**
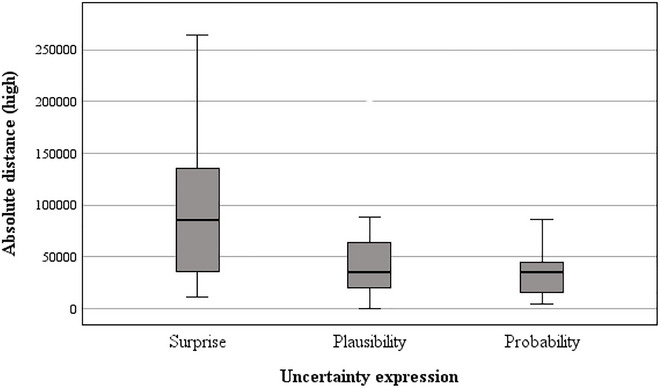
Absolute difference between baseline and *high* estimates for UK house prices by uncertainty expression.

To explore our findings further, univariate ANCOVAs were also run for each domain to test for the effect of uncertainty expression and cluster complexity on the absolute difference between the baseline value and participants’ high and low estimates separately, controlling for comprehension score. For absolute difference (high) estimates (see Figures 5 and 6), the covariate was nonsignificant in both domains (COVID‐19: *p* = 0.261, house price: *p* = 0.488). In both domains, there was a significant main effect of uncertainty expression (COVID‐19: *F*[2, 263] = 3.68, *p* = 0.027, η_p_^2^ = 0.03, house price: *F*[2, 263] = 16.96, *p* < 0.001, η_p_^2^ = 0.12).

Post hoc tests revealed that in both domains, the absolute difference of estimates from the baseline value was farther upwards in the *surprising* condition compared to both the *plausible* (COVID‐19: *t*[88] = 1.84, *p* = 0.035, *d* = 0.28, house price: *t*[88] = 3.97, *p* < 0.001, *d* = 0.60) and *probable* conditions (COVID‐19: *t*[88] = 2.09, *p* = 0.020, *d* = 0.32, house price: *t*[88] = 4.85, *p* < 0.001, *d* = 0.73). There was no significant difference between the *plausible* and *probable* conditions in either domain (COVID‐19: *t*[88] = 1.24, *p* = 0.108, *d* = 0.19, house price: *t*[88] = 1.38, *p* = 0.085, *d* = 0.21).

**FIGURE 7:**
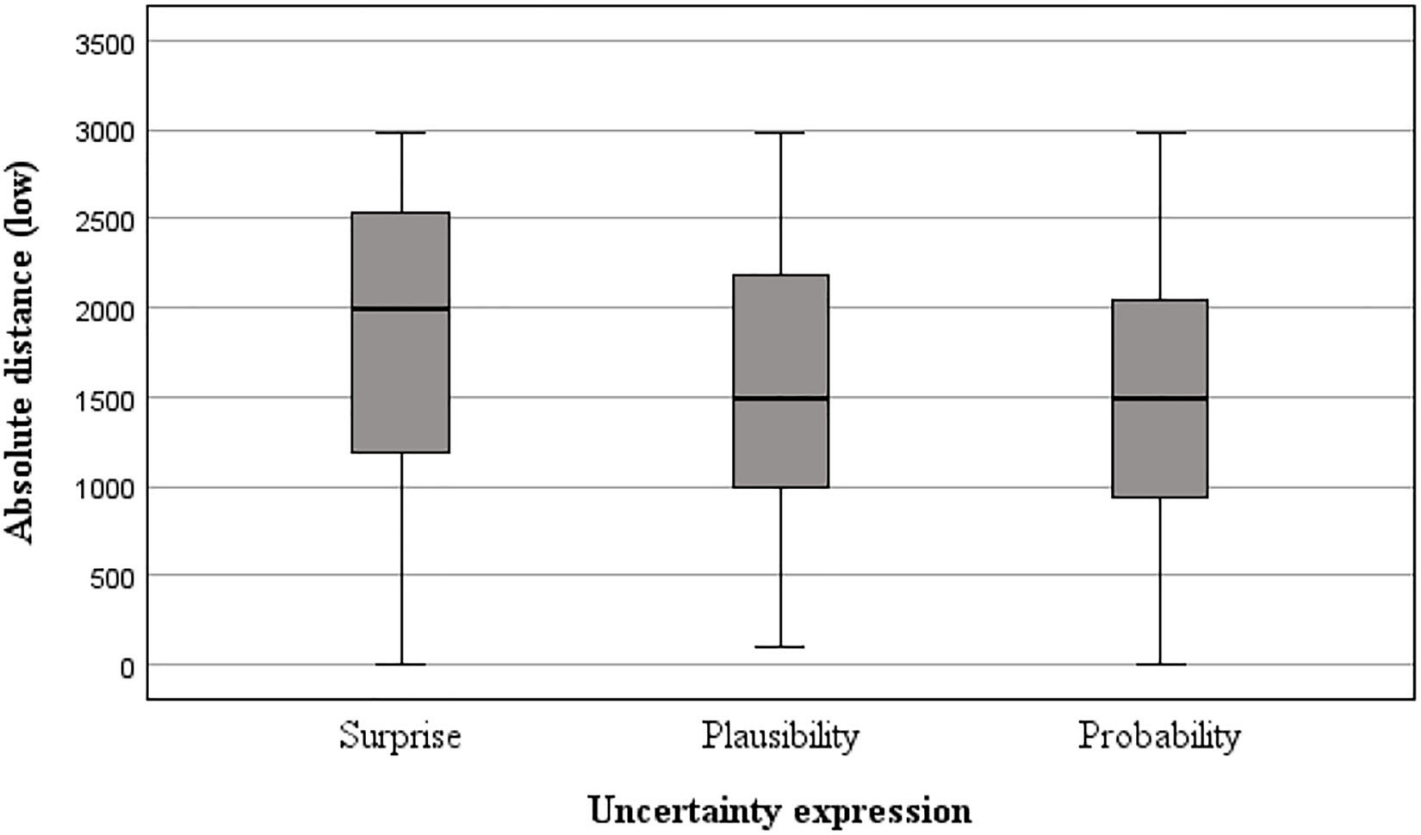
Absolute difference between baseline and *low* estimates for COVID‐19 deaths by uncertainty expression.

**FIGURE 8:**
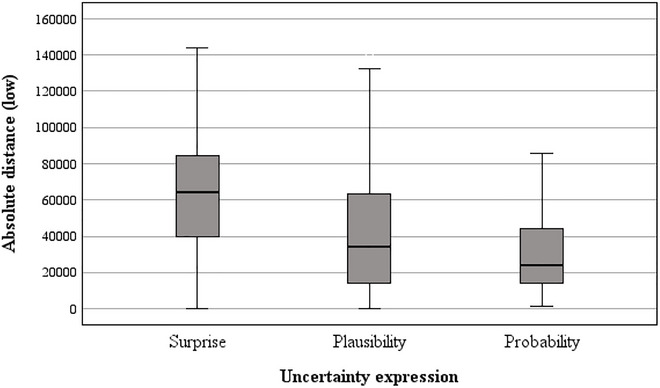
Absolute difference between baseline and *low* estimates for UK house prices by uncertainty expression.

The main effect of cluster complexity on absolute difference (high) estimates was nonsignificant in both domains (COVID‐19: *F*[1, 263] = 1.40, *p* = 0.239, η_p_^2^ = 0.005, house price: *F*[1, 263] = 2.36, *p* = 0.126, η_p_^2^ = 0.009), and no significant interaction was found between uncertainty expression and cluster complexity. For absolute difference (low) estimates (see Figures 7 and 8), the covariate was again non‐significant (COVID‐19: *p* = 0.521, house price: *p* = 0.891). The main effect of uncertainty expression was significant in the House price domain (*F*[2, 263] = 19.69, *p* < 0.001, η_p_^2^ = 0.13) but not the COVID‐19 domain (*F*(2, 263) = 2.14, *p* = 0.120, η_p_^2^ = 0.016).

Post hoc tests for house price domain revealed that the absolute difference of estimates from the baseline value was significantly farther downward in the *surprising* condition compared to both the *plausible* (*t*[88] = 4.01, *p* < 0.001, *d* = 0.60) and *probable* conditions (*t*(88) = 6.19, *p* < 0.001, *d* = 0.93). In addition, the absolute difference of estimates from the baseline value was significantly farther downwards in the *plausible* condition compared to the *probable* condition (*t*(88) = 1.81, *p* = 0.036, *d* = 0.27).

There was no significant main effect of cluster complexity on absolute difference (low) estimates in either domain (COVID‐19: *F*[1, 263] = 1.59, *p* = 0.208, η_p_^2^ = 0.006, house price: *F*[1, 263] = 0.011, *p* = 0.92, η_p_^2^< .01), and, again, no significant interaction was found between uncertainty expression and cluster complexity.

#### 7.4.1 Overall Direction of Movement From Baseline

**FIGURE 9:**
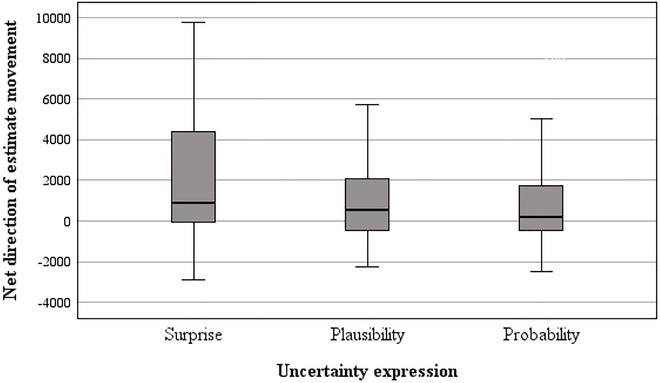
Net direction of estimate movement for COVID‐19 deaths by uncertainty expression.

**FIGURE 10:**
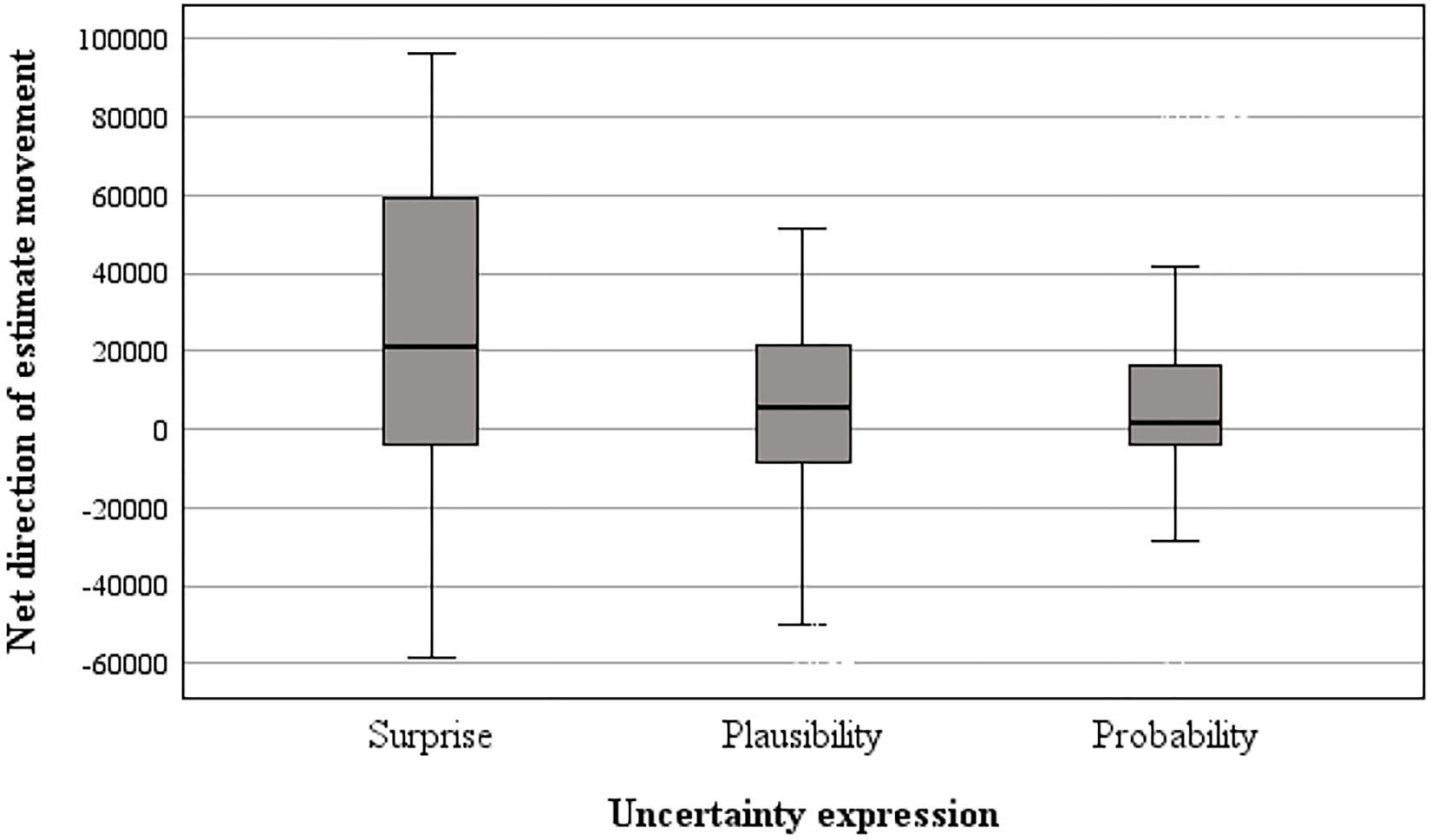
Net direction of estimate movement for UK house prices, by uncertainty expression.

Lastly, we explored whether participants’ estimates moved farther up or down from the baseline value overall, depending on whether they were asked to think about “surprising,” “plausible,” or “probable” values. Separate univariate ANOVAs were run for each domain. Uncertainty expression and cluster complexity were the between‐subjects factors, and comprehension score was entered as a covariate. The dependent measure (net direction of estimate movement) was created by subtracting the absolute difference between the baseline value and low estimate from the absolute difference between the baseline value and high estimate. Positively signed values are indicative of participants moving up above the baseline value, whereas negatively signed values are indicative of movement to below the baseline (see Figures 9 and 10).

In both domains, the covariate was non‐significant (COVID‐19: *p* = 0.241, house price: *p* = 0.358). There was a significant main effect of uncertainty expression in both domains (COVID‐19: *F*[2, 263] = 3.35, *p* = 0.037, η_p_^2^ = 0.03, house price: *F*[2, 263] = 6.58, *p* = 0.002, η_p_^2^ = 0.05), and the main effect of cluster complexity was nonsignificant (COVID‐19: *F*[1, 263] = 1.62, *p* = 0.204, η_p_^2^ = 0.01, house price: *F*[1, 263] = 3.72, *p* = 0.055, η_p_^2^ = 0.01). The uncertainty expression by cluster complexity interaction was borderline statistically significant for the COVID‐19 domain (*F*[2, 263] = 3.08, *p* = 0.048, η_p_^2^ = 0.02) but was nonsignificant for the house price domain (*F*(2, 263) = 1.10, *p* = 0.333, η_p_^2^ = 0.01).

Post hoc tests revealed that for both domains, in the *surprise* condition, the net direction of estimate movement was significantly farther above the baseline value compared to the *plausible* (COVID‐19: *t*[92] = 1.75, *p* = 0.042, *d* = 0.26, house price: *t*[132] = 2.66, *p* = 0.004, *d* = 0.40) and *probable* conditions (COVID‐19: *t*[88] = 1.99, *p* = 0.025, *d* = 0.30, house price: *t*[112] = 2.96, *p* = 0.002, *d* = 0.45). In both domains, there was no significant difference in the net direction of estimate movement between the *plausible* and *probable* conditions (COVID‐19: *t*[123] = 1.21, *p* = 0.115, *d* = 0.19, house price: *t*[174] = 0.26, *p* = 0.400, *d* = 0.04).

#### 7.4.2 Exploring Individuals’ Understanding of Surprise, Plausibility, and Probability

Separate analyses were carried out for the three uncertainty expression versions of the exit questionnaire. Specifically, a principal component factor analysis was conducted on the 11 items with oblique rotation (direct Oblimin with Kaiser Normalization). We would expect to see positive loading on the items that, according to the past literature, relate to the uncertainty expression that participants were asked about. In other words, we would expect to observe separate factors emerging, representing the three different concepts, that is, surprise, plausibility, and probability.

#### 7.4.3 Surprise Questionnaire

The Kaiser–Meyer–Olkin measure verified the sampling adequacy for the analysis, KMO = 0.92 (minimum acceptable value = 0.50). An initial analysis was run to obtain eigenvalues for each factor in the data. Two factors had eigenvalues over Kaiser's criterion of 1 and in combination explained 64.43% of the variance. The scree plot's point of inflection also suggested that two factors should be extracted. Table 3 shows the factor loadings after rotation. The items that cluster on each factor suggest that Factor 1 can simply be labeled “unsurprising” (essentially, probable and plausible combined) and Factor 2 can be labeled “surprising” (unexpected based on prior knowledge and experience).

**TABLE 3:** Results from a principal components analysis of the exit questions for participants in the surprise condition.

	Factor loading	
Exit questionnaire item	1	2
Compatible with your prior knowledge and experience	**0.857**	0.015
Likely to happen	**0.784**	−0.087
Justifiable in terms of the coherence of the causal relationships leading to it	**0.760**	0.098
Deviates widely from an expected outcome	−0.353	**0.616**
There is strong evidence that it will happen	**0.876**	−0.014
More likely to happen than not	**0.706**	−0.008
Contrasts with your prior knowledge and experience	−0.266	**0.590**
Follows from an understandable and compelling story	**0.837**	0.041
Has happened many times before	**0.847**	−0.011
There is nothing that might block or prevent it happening	**0.808**	−0.075
Another outcome was strongly expected	0.244	**0.841**

*Note*: The extraction method was principal component analysis with an oblique rotation (Oblimin with Kaiser Normalization). Factor loadings over 0.40 are in bold.

#### 7.4.4 Plausibility Questionnaire

The Kaiser–Meyer–Olkin measure verified the sampling adequacy for the analysis, KMO = 0.67. An initial analysis was run to obtain eigenvalues for each factor in the data. Three factors had eigenvalues over Kaiser's criterion of 1 and in combination explained 57.74% of the variance. The scree plot's point of inflection was ambiguous but consistent with the eigenvalues. Table 4 shows the factor loadings after rotation. The items that cluster on each factor suggest that Factor 1 can be labeled “probable” (likely to happen based on available evidence and prior knowledge), Factor 2 can be labeled “surprising” (unexpected based on prior knowledge and experience), and Factor 3 can be labeled “plausible” (causally coherent and with a compelling story and no blockers).

**TABLE 4:** Results from a principal components analysis of the exit questions for participants in the plausibility condition.

	Factor loading		
Exit questionnaire item	1	2	3
Compatible with your prior knowledge and experience	**0.396**	0.209	0.234
Likely to happen	**0.803**	0.059	−0.073
Justifiable in terms of the coherence of the causal relationships leading to it	0.045	−0.292	**0.582**
Deviates widely from an expected outcome	−0.034	**0.793**	−0.158
There is strong evidence that it will happen	**0.834**	−0.075	−0.058
More likely to happen than not	**0.701**	−0.162	−0.069
Contrasts with your prior knowledge and experience	0.074	**0.857**	0.011
Follows from an understandable and compelling story	−0.155	0.127	**0.771**
Has happened many times before	**0.737**	0.041	0.092
There is nothing that might block or prevent it happening	0.167	−0.079	**0.709**
Another outcome was strongly expected	−0.073	**0.787**	0.043

*Note*: The extraction method was principal component analysis with an oblique rotation (Oblimin with Kaiser Normalization). Factor loadings over 0.40 are in bold.

#### 7.4.5 Probability Questionnaire

The Kaiser–Meyer–Olkin measure verified the sampling adequacy for the analysis, KMO = 0.70. An initial analysis was run to obtain eigenvalues for each factor in the data. Four factors had eigenvalues over Kaiser's criterion of 1 (although factor four had an eigenvalue of 1.01) and in combination explained 67.92% of the variance. The scree plot's point of inflection was ambiguous and supported the extraction of either three or four factors. Two of Factor 4's items were shared (> 0.3) with Factor 3.^8^ For the purposes of the present analysis and parsimony, three factors were extracted. Table 5 shows the factor loadings after rotation. The items that cluster on each factor suggest that Factor 1 can be labeled “probable” (likely to happen based on available evidence), Factor 2 can be labeled “plausible” (happened many times before, compatible with experience, causally coherent and a compelling story with no blockers), and Factor 3 can be labeled “surprising” (unexpected based on prior knowledge and experience).

**TABLE 5:** Results from a principal components analysis of the exit questions for participants in the probability condition.

	Factor loading		
Exit questionnaire item	1	2	3
Compatible with your prior knowledge and experience	**0.721**	−0.053	0.177
Likely to happen	−0.027	**0.873**	−0.033
Justifiable in terms of the coherence of the causal relationships leading to it	**0.749**	0.116	−0.294
Deviates widely from an expected outcome	−0.018	0.026	**0.877**
There is strong evidence that it will happen	0.061	**0.596**	0.147
More likely to happen than not	−0.031	**0.821**	−0.091
Contrasts with your prior knowledge and experience	−0.040	0.156	**0.799**
Follows from an understandable and compelling story	**0.497**	0.255	0.195
Has happened many times before	**0.703**	−0.035	−0.021
There is nothing that might block or prevent it happening	**0.574**	−0.097	0.128
Another outcome was strongly expected	0.171	−0.182	**0.790**

*Note*: The extraction method was principal component analysis with an oblique rotation (Oblimin with Kaiser Normalization). Factor loadings over 0.40 are in bold.

## 8 Discussion

Scenario planning can be used to anticipate and prepare for potential extreme outcomes. Although extreme futures may be described in many ways, including as (highly) improbable, implausible, or surprising, in the standard, matrix‐based IL scenario‐planning approach, the focus is explicitly on what is plausible (Cairns and Wright 2018). While this chimes with the now recognized need for risk assessments to “go beyond probability” (Aven 2016), a question remains regarding the extent to which plausibility genuinely extends thinking beyond what it would be when exclusively using probability (Glette‐Iversen et al. 2022). In this paper, we suggest that thinking in terms of what is surprising can stretch and challenge existing mindsets. Unlike both plausibility and probability, surprise focuses on the contrast between potential future outcomes and both past experiences and present knowledge (Teigen and Keren 2003; Urueña 2019).

In this paper, we employed an experimental approach to examine how thinking about surprise rather than probability or plausibility may better stimulate consideration of extremes. We compared the values assigned to the resolved outcomes of the clustered driving forces created in the early stages of IL scenario planning with an assigned value representing a baseline, “business‐as‐usual” outcome. We did this for two different domains—that is, the number of COVID‐19 deaths in England and median house prices in the United Kingdom.

In both domains studied, we observed the hypothesized main effect of uncertainty expression on the *absolute range in* participants’ estimates (H1) and found that the absolute range in estimates was significantly greater in the “surprising” condition than in either the “plausible” condition (H1a) or the “probable” condition (H1b). In other words, under both probable and plausible expressions, individuals’ estimates of COVID‐19 deaths (in England) and median (United Kingdom) house prices fell closer to the baseline value provided for each domain, compared to when they were prompted to think of surprising outcomes. In addition, although we found support for the prediction that the absolute range in estimates would be greater in the “plausible” than “probable” condition (H1c), this was only the case for the house price domain.

Exploratory analyses revealed that, in both domains, the high estimates moved farther upward from the baseline value under the “surprising” condition compared to the “plausible” and “probable” conditions, while there was no significant difference between the latter two conditions. This pattern was partially mirrored for the low estimates, where movement farther downward from the baseline under the “surprising” condition was significantly greater than the other two conditions in the house price domain, with there being no such effect for the COVID‐19 domain. However, the range of possible values is naturally truncated at zero in both domains, which should be recognized when considering the difference in the effect on low and high estimates. Considering this natural truncation, we might expect the high estimate to move farther from the baseline. Finally, it was revealed that, for both domains, the direction of movement away from the baseline value was significantly farther upward overall under the “surprising” condition than the other two conditions, while there was no significant difference between the “plausible” and “probable” conditions.

The principal components factor analyses revealed that when asked about the meaning of an uncertainty expression (i.e., surprise, plausibility, and probability), participants appropriately highlighted items from the literature referring to each of these concepts. Of note, although all participants were able to clearly differentiate surprise from plausibility and probability, some participants demonstrated less differentiation between the latter two concepts. This reduced ability to discriminate between plausibility and probability is compatible with the findings pertaining to the main part of the experiment summarized above—that is, only partial support for H1c and the exploratory analyses regarding movement of (high and low) estimates from the baseline, as well as overall direction of movement away from the baseline showing no significant difference between the plausible and probable conditions.

Together, these findings suggest that, in the minds of individuals, plausible futures do not tend to differ significantly from probable ones. Both these concepts focus expectations about the potential extremity of the future in a similar way based on past occurrences and presently available knowledge. This is compatible with the conclusion reached by Glette‐Iversen et al. (2022), who recently reviewed research on plausibility: that the concept of plausibility should be seen as a measure of uncertainty capturing a combination of likelihood and judgments based on and about the supporting knowledge.

In scenario planning, plausibility may thus lead to the production of scenarios that are conservative, and which do not assist in creating an expectation for surprises or in anticipating them. Thinking in terms of surprise (rather than plausibility or probability) fosters consideration of a broader range of outcomes. In addition, thinking in terms of surprise can help stretch imagination of what is possible beyond present knowledge and past outturns.

We also hypothesized that more complex clusters of driving forces would generate a wider absolute range in estimates than simpler clusters (H2). In setting out this hypothesis, we argued that cluster complexity could conceivably cut both ways in its effect. On the one hand, the more intermediate causes it captures, the greater the uncertainty surrounding a cluster's resolved outcome, and therefore, the wider the range of its potential values. On the other hand, cluster complexity could temper the effect of the three uncertainty expressions and reduce the differences between them because a greater number of intermediate causes may be perceived as increasing the possibility for compounded second‐order effects and positive feedback, which may take the resolved outcome far beyond its historical values. If a participant successfully traces through the complex causal logic, she may, therefore, assign more extreme values to its resolved outcome under all three expressions.

However, we also suggested that contrary to H2, we might just as easily expect a greater difference between the effects of the three uncertainty expressions from a simpler cluster of driving forces, with the greatest difference being between “surprising” and the other two expressions (i.e., plausible and probable). This is because greater cluster complexity may make it more difficult to trace through causal logic, leading to a falling back on what is already known based on past occurrences. This might mean that the “surprising” expression will move further from the baseline value compared to the other two expressions, which would move little.

However, contrary to these conjectures, our findings did not detect an effect from cluster complexity. This may have been because these various effects, which we stated could cut both ways, did exactly that. These effects related to cluster simplicity/complexity may have offset and negated each other. Alternatively, it could simply be that cluster complexity does not have any effect. Determining the true nature of any effect from cluster complexity might require additional experimentation that is more directly targeted at uncovering that effect in isolation.

More generally, our experiment was based on a simplified set of stimuli compared to that participants would be subjected to within a real IL scenario process. In practice, an IL scenario process might last several days and be conducted in an in‐person and group‐based workshop setting, rather than online. There would be social stimuli from interaction with other participants, which does not feature anywhere in our experiments. The impact on changing participants’ mindset might, therefore, be considered an emergent effect of the whole process rather than one part of it. Given our experimental setup, our research emphasizes the individual‐focused parts of the process. This might reduce the external validity and generalizability of the present findings (Derbyshire, Dhami, et al. 2023) and may raise a question about the appropriate unit of analysis for research on scenario planning (i.e., group vs. individual). However, future research focusing on the group ought to consider the individual‐level effect on group consensus, and our work contributes some potential insights in that regard (e.g., how far does a group shift from the extreme values that individual members bring to the “table” if they are prompted to think in terms of surprise? And, what factors might affect this?).

Another issue to note is that the origins of the IL scenario process lie in assisting businesses to anticipate disruptive changes as part of their strategic decision‐making processes (Bradfield et al. 2005; Derbyshire 2023; Healey and Hodgkinson 2008, 2024; Schoemaker 1993). In contrast, we are here considering its use to anticipate potentially extreme outcomes in the context of a risk assessment. In this latter context, its use by an individual rather than in a group‐based setting might be more likely. Adapting its use to that context might ultimately require several changes to the standard IL process. That tailoring must begin with testing its effect when used individually. Other changes might include a more direct focus on events and their outcomes. We encourage follow‐up research that tailors the IL scenario process into a tool for “going beyond probability” (Aven 2016) in a risk assessment context.

Finally, as Derbyshire, Dhami, et al. (2023) also discussed, the development of scenario planning has been held back by a paucity of empirical research that can identify the most appropriate methods and techniques to use for its implementation. Part of the reason for this paucity of empirical research is the complexity of conducting experiments in ever‐changing social settings that make experiments difficult to implement and replicate. Progressing the field by increasing the amount of empirical research conducted within it requires compromises. Online experiments are one way to accelerate empirical research on scenario planning. Inevitably, each such individual‐level experiment will foreground some aspects of the scenario process at the expense of underrepresenting others. For that reason, it is the weight of evidence across many studies that matters and not the findings of any one study alone (Derbyshire, Dhami, et al. 2023). Our research has contributed to the weight of evidence on scenario planning and its effect, and we look forward to reading others that do similarly.

Given that our research focused on one element of the IL scenario process (i.e., that prompting participants to assign extreme values to clustered sets of driving forces) and aimed to study individual psychological responses to alterations in that one element, we believe the adopted methodology to have been appropriate, especially given the practical challenges associated with conducting behavioral research during the pandemic. This is not to say, however, that future research should not attempt to replicate our findings using an alternative approach, such as what might be termed a “full‐blown scenario exercise” in which the study participants carry out all elements of the IL scenario process. It should be borne in mind, however, that this would require several hundred motivated and knowledgeable participants grouped into equivalent teams who each work on a common scenario exercise that is equally relevant to all of them over an extended period (i.e., the several days it takes to complete an in‐person IL scenario exercise). Anything less would make the findings potentially statistically unreliable. The considerable (or even insurmountable) practical difficulties this would entail are perhaps the main reason for the current paucity of empirical research on scenario planning. Considering this, online studies conducted on individual elements or parts of the scenario process are not only perfectly valid but an essential means by which to increase the amount of empirical research on this widely used but undertested tool.

## 9 Conclusion

The field of risk analysis has, for some time now, recognized the need to reduce susceptibility to surprises by taking full account of uncertainty and going beyond probability as part of a comprehensive risk assessment. Yet, governments, businesses, and other organizations―some of which carry out seemingly comprehensive risk assessments―keep getting caught out by so‐called “surprises,” some of which could have been anticipated. Until now, while the need to reduce susceptibility to surprises by taking full account of uncertainty and going beyond probability is widely recognized, how this need might be met within a risk assessment has remained an open question, which we have addressed directly and empirically.

On the face of it, plausibility‐based scenario methods like IL provide one means by which to meet this need. Surprises result from a contrast between the extremity of an empirical observation and an individual's pre‐existing representation of the range of potential extremity, the bounds of which the empirical observation has exceeded. The avoidance of surprises must therefore involve stretching this representation of the range of potential extremity so that it captures the full extent of the future's uncertainty. That is what plausibility‐based scenario planning is designed to do. Yet, as highlighted herein, several studies on plausibility have suggested a problem with this logic: plausibility embeds thinking in past experiences, presently available knowledge, and prior outturns in the same way that probability in both its objective and subjective forms does. Individuals explicitly look to their past experiences as a frame of reference when making judgments about plausibility. Indeed, this paper has evidenced that participants in a simulated IL scenario‐planning exercise assign a similar range of values to the resolved outcome of clustered sets of driving forces when prompted using the expression “plausible” and when prompted using the term “probable.” This suggests individuals perceive plausibility and probability synonymously. Given this, plausibility‐based scenario planning might not only fail to stretch thinking beyond what it would be when using probability; it might even exacerbate the tendency to focus on past experiences and outturns and to draw on presently available knowledge. In which case, it cannot assist risk assessors to “go beyond probability” so as to take full account of uncertainty and reduce susceptibility to surprises.

However, by adapting the IL scenario method in the simple way outlined in this paper, it can stimulate consideration of a wider range of potential outcomes, increasing the chance that extreme future outturns fall within the considered range—and in so doing, susceptibility to surprises. By using the expression “surprising” as the prompt for assigning values to the resolved outcomes of clustered sets of driving forces within IL scenario planning, a wider range of outcomes is assigned than that assigned using either “plausible” or “probable” as a prompt for uncertainty expression. This increases the chance that the future's full potential extremity might be captured within the range, thereby reducing the potential for surprises.

## Data Availability

The data that support the findings of this study are available on request from the corresponding author. The data are not publicly available due to privacy or ethical restrictions.

## Endnotes

1 We follow the understanding of “knowledge” and “strength of knowledge” set out in Derbyshire and Aven (2025, 1199), in which knowledge is a “belief about a focal matter that is justified by a set of evidence, which may include data and information of all types, but which could also include evidence in the form of testing, argumentation, modelling, or simulation” (Aven and Flage, 2023). Based on Derbyshire and Aven's (2025, 1199) understanding of it, the strength of knowledge therefore refers to the “strength of the beliefs forming the knowledge and the supporting evidence” (Aven, 2016; Aven and Flage, 2023). It reflects both the quality of the evidence and how well the beliefs are supported by the evidence. The completeness of the evidence is an aspect of the strength of knowledge concept (Derbyshire and Aven, 2025, 1199).

2 The experiment was conducted in August 2021, which was approximately 3 months after the end of the second wave of coronavirus in the United Kingdom, and just before the start of a potential third wave in Autumn/Winter 2021/2022.

3 While this very explicit reference to plausibility is a specific feature of the Cairns and Wright (2018) version of IL, there are several different versions of IL and in some consideration of plausibility may be more implicit than explicit. Nevertheless, whether implicitly or explicitly, consideration of plausibility is integral in all versions.

4 See Derbyshire, Feduzi, et al. (2023) for a discussion of how to assess uncertainty as part of this ranking exercise. The distinction between risk and uncertainty originally made by Knight (1921) is often used to justify the use of scenario planning instead of more mainstream decision‐making aids. The logic is that scenario planning can take account of uncertainty that is irreducible to risk in a way that these more mainstream aids cannot because scenario planning is based on plausibility and not probability. However, as alluded to in the paper by Derbyshire and Aven (2025) that establishes the groundwork for this one, this distinction between risk and uncertainty, which is prevalent in some parts of economics (e.g., post‐Keynesian economics), has been made redundant in the field of risk analysis by recent developments. The framework set out in Derbyshire and Aven (2025) is designed to do away with Knight's (1921) distinction between risk and uncertainty, which Kay and King's (2020) concept of “radical uncertainty” is based on. Derbyshire and Aven's (2025) framework accommodates all uncertainties of any type, making Knight's (1921) distinction redundant. Moreover, Knight's (1921) distinction was between circumstances in which an “objective” probability can be determined and circumstances in which it cannot be. A subjective probability can be assigned to anything. Note, however, that subjective probability and Bayesian inference are not the same thing. The Bayesian approach may well be a form of subjective probability, but it is not a particularly useful one for assessing surprises, as its axioms require the closure of the future through the ex‐ante listing of all its possibilities. In short, the field of risk analysis has moved beyond Knight's (1921) distinction. What is emerging instead in the field of risk analysis is an “uncertainty‐based risk perspective” (Aven, 2025), which recognizes that uncertainties can also come in the form of unknowns, surprises, and extremes. This shift in perspective makes testing scenario planning's effect even more imperative. If risk analysis is shifting away from its traditional emphasis on averages and the stable moments of known probability distributions (Aven, 2025), then there is great potential for scenario planning to become one of its most important tools, but only if it can take account of the full extent of uncertainty in a way traditional risk‐analysis approaches cannot.

5 Schoemaker (1993) and Kuhn and Sniezek (1996) are the two that are most directly related to this one, but they are by no means the only empirical studies of scenario planning and its effect. Readers are referred to Goodspeed (2020, 152–156) for a list of several other empirical studies that have taken place across several domains. An important study not included in that list, however, is that by Phadnis et al. (2015). For a more recent study conducted in a strategic‐management context, see Healey and Hodgkinson (2024).

6 Comparable studies exploring the effect of question wording on uncertainty estimates have found broadly medium‐sized effects (e.g., Mandel et al. 2020; Speirs‐Bridge et al. 2010).

7 Data were collected in September 2021 and so the baseline values given were for August 2021. Participants were asked to estimate future values 6 months (COVID‐19) or 12 months (house prices) from the date of the baseline values.

8 Oblique rotation is considered preferable to orthogonal rotation when it is believed that factors are likely to be related (as is the case here). Rotation is included because otherwise most variables tend to have high loadings on the most important factor and small loadings on all other factors. Rotation is used to discriminate better between factors. It involves rotating the axes so that variables are loaded maximally to only one factor.

## Appendix 1

Participants were presented with the following stimuli.

### COVID‐19 Deaths in England Domain

Complex cluster—High outcome

Complex cluster—Low outcome

Simple cluster—Neutral

*Baseline outcome value*—COVID‐19 deaths recorded in England in August 2021 = 2989 (*Deaths within 60 days of a positive COVID‐19 test or where COVID‐19 is mentioned on the death certificate*)

Simple cluster—High outcome

Simple cluster—Low outcome

*COVID‐19 deaths in England domain*: The following questions appeared after each NEUTRAL cluster (with the first six used to assess participants’ comprehension, and question 7 used as a manipulation for the cluster complexity variable):

1. How many causal connections do you see in the cluster? [numerical response]
2. How many *common causes* do you think there are in the cluster (where one cause leads to two or more effects)? [numerical response]
3. How many *common effects* do you think there are in the cluster (where two or more causes lead to one effect)? [numerical response]
4. Which do you think is the most important item (box) in the cluster? [Drop‐down list]
5. [Question 5a was used with the *complex* cluster and Question 5b was used with the *simple* cluster:]

a. As travel to foreign countries increases, what will happen to the number of cases? [options: (a) it will increase, (b) it will decrease, (c) it will stay the same]
b. As social interaction increases, what will happen to the number of cases? [options: (a) They will increase, (b) They will decrease, (c) They will stay the same]

6. [Question 6a is was used with the *complex* cluster and Question 6b was used with the *simple* cluster:]

a. If a vaccine‐resistant strain does emerge, how will that effect the level of immunity provided by existing vaccines? [options: (a) It will increase, (b) It will decrease, (c) It will stay the same]
b. If the level of immunity provided by existing vaccines decreases, what may happen to the level of hospitalization? [options: (a) It will increase, (b) It will decrease, (c) It will stay the same]

7. Overall, how complex is this cluster? [scale: 1 = extremely simple to 7 = extremely complex]
8. In your opinion, are there any key factors (causes or effects) missing from the cluster? [open response]

The following question appeared after each high and low (complex/simple) cluster:

Based on the causal logic of the cluster and the baseline value of 2989, please give a [surprising/plausible/probable] [high/low] value for the number of COVID‐19 deaths that will be recorded in England in February 2022.

## Appendix 2

Participants were asked to fill in the following exit questionnaire upon completion of their tasks.

### Exit Questionnaire

To what extent do you agree with the following statements? Please score each statement from 0 to 10, where 0 equals “not at all” and 10 equals “completely.”

(Note: the questions were presented in a random order.)

An outcome is [surprising/plausible/probable]:

1. If it is compatible with your prior knowledge and experience.
2. If it is likely to happen.
3. If it is justifiable in terms of the coherence of the causal relationships leading to it.
4. If it deviates widely from an expected outcome.
5. If there is strong evidence that it will happen.
6. If it is more likely to happen than not.
7. If it contrasts with your prior knowledge and experience.
8. If it follows from an easily understandable and compelling story.
9. If it is strongly expected to happen.
10. If there is nothing that might block or prevent it from happening.
11. If another outcome was strongly expected.
